# Comparative Genomics of *Staphylococcus rostri*, an Undescribed Bacterium Isolated from Dairy Mastitis

**DOI:** 10.3390/vetsci10090530

**Published:** 2023-08-22

**Authors:** Desiree Corvera Kløve, Michael Farre, Mikael Lenz Strube, Lærke Boye Astrup

**Affiliations:** 1 Department of Health Technology, Technical University of Denmark, Kemitorvet, 2800 Kongens Lyngby, Denmark; 2 SEGES Innovation P/S, Agro Food Park, 8200 Aarhus, Denmark; 3 Department of Biotechnology and Biomedicine, Technical University of Denmark, Søltofts Plads, 2800 Kongens Lyngby, Denmark

**Keywords:** bovine mastitis, non-aureus Staphylococci (NAS), *Staphylococcus rostri*, whole genome-sequencing (WGS), phylogeny, virulence factors, antibiotic resistance

## Abstract

**Simple Summary:**

Mastitis is one of the most common diseases in dairy cows. To control mastitis in dairy cows, it is vital to understand the causative agents including their respective mode of action. Several bacterial species may cause bovine mastitis although non-aureus Staphylococci (NAS) are often reported as the most frequently observed cause of subclinical mastitis in dairy cows. As species of NAS may differ in their epidemiology and pathogenicity, performing diagnostics at the species level is crucial. This study is about *Staphylococcus rostri*, which is a newly identified NAS. *Staphylococcus rostri* is rarely reported or characterized in domestic animals, and, to our knowledge, never as a causative agent of dairy mastitis. With the present study, we report the finding of 81 *Staphylococcus rostri* isolates from nine dairy herds and mainly from subclinical mastitis. We characterized these *S. rostri* isolates with whole genome sequencing analysis. The results showed a limited distribution of known genes associated with virulence (*clpP* and *clpC*, *n* = 81 isolates) and antibiotic resistance (*str*, *n* = 1 isolate and *lnuA n* = 2 isolates). However, we found that the *S. rostri* isolates consisted of herd-specific clones, indicating that each herd had separate introduction source(s). Overall, this study suggests that *S. rostri* acts as a mastitis pathogen, despite the exact pathogenetic mechanisms of *S. rostri* still requiring full characterization and remaining unknown.

**Abstract:**

This study characterizes 81 *S. rostri* isolates from bovine mastitis (of which 80 were subclinical). The isolates were first identified as *S. microti* by MALDI-TOF MS, but later whole genome sequencing analysis allowed reclassification as *S. rostri.* The isolates were derived from 52 cows and nine dairy herds in Denmark. To describe the pathogenicity of *S. rostri*, we used whole genome sequencing to infer the distribution of genes associated with virulence, antibiotic resistance, and mobile genetic elements. Also, we performed a core-genome phylogeny analysis to study the genetic relatedness among the isolates. All 81 isolates expressed the same virulence profile comprising two putative virulence genes, *clpP* and *clpC*. Three isolates carried a resistance gene encoding streptomycin (*str*) or lincomycin (*lnuA*) resistance. The distribution of plasmids suggested the detected antibiotic resistance genes to be plasmid-mediated. Phages were abundant among the isolates, and the single isolate from clinical mastitis acquired a phage disparate from the rest, which potentially could be involved with virulence in *S. rostri*. The core genome phylogeny revealed a strong genetic intra-herd conservation, which indicates the source of introduction being herd-specific and might further imply the ability of *S. rostri* to adapt to the bovine niche and spread from cow-to-cow in a contagious manner. With this study, we aim to acquaint clinicians and professionals with the existence of *S. rostri* which might have been overlooked so far.

## 1. Introduction

Mastitis is one of the most common diseases in dairy cows, and Staphylococci are key agents in this regard [1]. In relation to bovine mastitis, *Staphylococcus aureus* is considered one of the major pathogens owing to its frequency and severity as a mastitis agent. All the non-aureus Staphylococci (NAS) are oppositely referred to as minor pathogens since they are primarily associated with subclinical (SCM) or mild clinical mastitis (CM). In terms of numbers, however, NAS are reported as the most prevalent mastitis-associated bacteria causing increased somatic cell counts (SCCs) despite the absence of severe infection. Thereby, NAS can negatively affect the milk quality and subsequently comprise a burden to milk production [2,3]. 

Traditionally, NAS has been considered a homogeneous group; hence, species differentiation has not been a routine procedure at diagnostic laboratories or in research. More recent research, however, demonstrates that species-specific differences among NAS exist, e.g., regarding their epidemiology, virulence factors, and antibiotic susceptibility. Therefore, it is now recommended to consider NAS as a heterogeneous group, which starts with studying each member individually and accordingly conducting diagnostics on species level [4,5]. Conventional mastitis diagnostics rely on culture and phenotypic characterization often based on commercial biochemical kits. Usage of such diagnostics is known to be laborious, time-consuming, and (most importantly) often fails to distinguish among many mastitis bacteria, including the NAS species, subsequently failing to provide correct diagnoses [6]. Increased application of molecular methods and matrix-assisted laser desorption ionization–time of flight mass spectrometry (MALDI-TOF MS) reduces such challenges, and when implemented in the laboratory, these techniques are generally considered faster, more cost-effective, and more reliable for the identification of mastitis bacteria on species level [6,7,8,9].

To date, more than 20 different NAS species have been isolated from bovine mastitis, including *S. chromogenes*, *S. simulans, S. haemolyticus, S. xylosus,* and *S. epidermidis* as the most frequently identified species [4]. On the contrary, *Staphylococcus microti* and *Staphylococcus rostri* are two examples of novel NAS members. They were first discovered in 2010 from common voles (*Microtus arvalis*) with Brucella infection and noses of healthy pigs, respectively [10,11,12]. While *S. microti* up till now has been reported in milk from bovine mastitis three times (Poland 2016, United States 2019, and Germany 2020) [5,13,14], *S. rostri* is only once encountered in the literature in association with bovine mastitis (United States 2019) [14], although *S. rostri* has been isolated related to dairy cows previously. First, in a single bulk-tank milk sample (Belgium 2017) [15], and second in fecal samples from healthy dairy cows (Belgium 2019) [16]. As such, findings of *S. microti* and *S. rostri* are rarely reported in the literature and characterization studies on these NAS species in relation to bovine mastitis remain few. This might suggest that these species are seldom found in the milk from bovine mastitis or alternatively that they have simply been overlooked hitherto (perhaps due to limited species differentiation by conventional diagnostic methods). 

The present study provides a genotypic description of 81 *S. rostri* isolates from bovine mastitis (mainly SCM) in Denmark. The isolates were initially identified as *S. microti* by MALDI-TOF MS followed by whole genome sequencing (WGS), which revealed that the isolates were misclassified as *S. microti* at first. The present characterization of *S. rostri* focuses on genetic relatedness among the isolates, and the presence of virulence genes, antibiotic resistance genes, and mobile genetic elements (MGEs). To our knowledge, this study comprises the first genotypic characterization of *S. rostri* in bovine mastitis.

## 2. Materials and Methods

### 2.1. Isolate Collection and Identification

Most of the isolates in this study (*n* = 80/81) originated from another research study focusing on bacteria associated with bovine SCM in Denmark, which was conducted at Centre for Diagnostics, Technical University of Denmark (CfD, DTU) (unpublished). In that study, quarter milk samples were collected during 2019 to 2020 from dairy cows with SCM, i.e., cows with a SCC of ≥200.00 cells/mL and no visible signs of infection. The samples were collected just after the monthly milk recording, performed by the national Registration and Milk Recording organization (RYK) which monitors all DHI herds in Denmark. The milk samples were classified as either new or persistent infections. We defined persistent infection as cows having had an increased SCC of ≥200.000 cells/mL measured at least two times during the last three consecutive and monthly RYK recordings. New infection was defined as cows having had one SCC ≥200.00 cells/mL measured for the last three RYK recordings. The last isolate (*n* = 1/81) in the present study, came from a CM milk sample, submitted to CfD, DTU in 2019 by a veterinary clinic for diagnostic analysis.

For all milk samples, 10 µL was cultured on blood agar with 5% calf blood (SSI Diagnostica A/S, Hillerød, Denmark) and incubated overnight at 37 °C. Pure subcultures were identified by MALDI-TOF MS (Bruker Daltonics, Bremen, Germany) as described by Astrup and associates, 2022 [6]. A MALDI-TOF MS score ≥2.00 was considered an accurate identification on the species level, a score between 1.70 and 1.99 as an accurate identification on the genus level, and a score ≤1.69 as a non-reliable identification [6,17], and in this study, such cases were classified as ‘No ID’. The identified isolates were stored in LB bouillon with 15% glycerol at −80 °C until further analysis. Isolates found in mixed cultures with >2 different bacterial species were classified as contaminated and omitted from the study [18]. 

### 2.2. Whole-Genome Sequencing Analysis

All isolates identified as *S. microti* by MALDI-TOF MS at CfD, DTU from 2019 to June 2020 were whole genome sequenced to perform the genotypic characterization. DNA extraction and sequencing were outsourced to Novogene (Novogene (UK) Co., Ltd., Cambridge, UK). In brief, DNA extraction was performed using an AllPrep DNA/RNA kit (Qiagen, Hilden, Germany) according to the manufacturer’s instructions. Extracted DNA was sequenced using the Illumina NovaSeq6000 platform in a 2 × 150 bp paired-end configuration. 

The quality of the raw reads was examined using FastQC (v.0.11.9). SPAdes (v.3.13.1) was used for genome assembly, applying the settings “-k 21,33,55,77 --careful” [19]. The assembled genomes were inspected using QUAST (v.5.0.2) [20]. Taxonomic identification was determined by average nucleotide identity (ANI) analysis of the assembled genomes and the three closest reference genomes: *S. microti* DSM 22147 (GenBank Accession no. GCA_002902635), *S. rostri* DSM 21968 (GenBank Accession no. GCA_002902145), and *S. muscae* ATCC 49910 (GenBank Accession no. GCA_003019275) [10,11] using Pyani with ANIb [21]. Data handling and visualization was conducted in R (v. 4.2.3) using the pheatmap package library [22].

Prokka (v. 1.14.6) was used for gene annotation using the *S. rostri* DSM 21968 as reference [23]. The GFF3 files generated by prokka were used by roary (v. 3.7.0) to define the core and accessory genomes (95% blastp cutoff) [24]. Here, the core genome consists of genes shared between all isolates, while the accessory genome is non-core genes carried by at least one isolate. Based on the core genome alignment, a phylogenetic tree was generated using RAxML-NG and the GTR + G model [25], which was then visualized in iTol [26]. The core genome alignment was further converted to a single-nucleotide polymorphism (SNP) matrix using snp-dist (v. 0.8.2) (https://github.com/tseemann/snp-dists (accessed on 2 June 2023)). The GFF3 files were additionally used with the query_pan_genome function with roary to study potential gene differences between sets of isolates [24]. Chi-square test was applied for the descriptive statistics in R (*p* < 0.05) [24].

PhiSpy (v. 4.2.21) was used to predict prophage sequences [27] and vContact2 (v. 0.11.3) to obtain clustering information of the identified prophages [28], which was finally mapped as a clustering network in Cytoscape (https://cytoscape.org/ (accessed on 8 May 2023)). The Abricate pipeline (https://github.com/tseemann/abricate (accessed on 21 April 2023)) was applied combined with VFDB (70% identity and coverage) [29], ResFinder (90% identity and 60% coverage) [30,31,32], and PlasmidFinder (95% identity and 60% coverage) [32,33] to investigate the presence of virulence, antibiotic resistance, and plasmid-associated genes. 

## 3. Results

### 3.1. Bacterial Species Delineation

In the period 2019 to June 2020, a total number of 81 isolates from bovine mastitis were identified as *S. microti* at the diagnostic laboratory of CfD, DTU. As *S. microti* is a NAS rarely encountered nor described in the literature, we performed WGS on the isolates to characterize this novel species. The mean assembly length and GC-content were 2.30 Mb and 38.47%, respectively (Table S1). 

To confirm the taxonomy of the isolates, an ANI analysis was performed including all 81 isolates and genomes of reference strains of *S. microti*, *S. rostri*, and *S. muscae* (Figure S1). These reference species were chosen due to previous studies demonstrating them to be the nearest relatives to each other [10,11,13]. The similarity of the 81 test isolates was found closest to *S. rostri* DSM 21968 with ANI values >99.0%, whereas the ANI values for *S. microti* DSM 22147 and *S. muscae* ATCC 49910 were ~83% and ~80%, respectively. Following the species boundary cut-off of >95% [21,34], the 81 isolates were taxonomy classified as *S. rostri* accordingly. 

To enable future comparative studies on *S. rostri*, we deposited all 81 genomes from this study to the National Center for Biotechnology Information (NCBI) under BioProject no. PRJNA988321.

### 3.2. S. rostri Isolate Distribution

Of the 80 *S. rostri* SCM isolates, 35 isolates were classified as new SCM and 45 as persistent SCM. The last isolate (*n* = 1/81) came from CM (Table S2). 

Overall, the isolates originated from 52 cows distributed among nine Danish dairy herds. Besides a single herd (herd 5) being located in Zealand, Denmark, all the other herds are distributed across Jutland, Denmark which represents the main geographical area of dairy herds in Denmark. The number of isolates among each herd varied from one isolate up to twenty-five isolates (Table 1). For 30 cows, *S. rostri* was detected in a single quarter, while 21 cows had *S. rostri* detected in two or three quarters. Finally, a single cow had *S. rostri* in all four quarters (Table 1). 

Twenty-four isolates (29.6%) were found in pure cultures and 57 isolates (70.3%) in mixed cultures, i.e., cultures containing two different bacterial species (Table S2). The CM isolate was found in pure culture, and no significant association was found between type of culture (pure/mixed) and type of mastitis (new SCM/persistent SCM) (*p* = 0.4741). 

The mixed cultures showed that *S. rostri* was identified in combination with 16 different bacterial species in total (Table 2). The most frequently observed species in the mixed cultures were other NAS (*n* = 34/57), including *S. simulans*, *S. epidermidis*, and *S. haemolyticus* (Table 2). These three NAS species are generally considered among the most prevalent ones causing bovine SCM and furthermore, they are often recognized as being involved with persistent SCM [4]. For the latter reason, we investigated if the occurrence of *S. rostri* in mixed culture with another NAS was associated with the type of mastitis (new SCM/persistent SCM), but no statistical significance was found (*p* = 0.2025). For all herds, a mixed culture containing *S. rostri* with another NAS, was found in a minimum of one case, and none of the NAS species were exclusively related to specific herds, i.e., *S. simulans, S. epidermidis*, and *S. haemolyticus*, etc., were identified from several herds. At cow-level, 22 cows had *S. rostri* detected in >2 quarters. Out of these twenty-two cows, only four cows showed the same pair of *S. rostri* + another NAS in >2 quarters; one cow had *S. rostri* + *S. chromogenes* in two quarters, one cow had *S. rostri* + *S. simulans* in two quarters, while two other cows had *S. rostri* in three quarters where either *S. epidermidis* or *S. simulans*, respectively, were detected in two of the same quarters (Table S2). For the remaining 18 cases of *S. rostri* in ≥2 quarters pr. cow and in mixed culture with another NAS, different NAS species were detected among each quarter. 

### 3.3. Pan-Genome Analysis

A pan-genome analysis was completed to deduce the genetic content and relatedness among the 81 isolates. For this, the porcine *S. rostri* DSM 21968 strain was included as a reference. This strain was selected due to it being the only reference genome of *S. rostri* available in the NCBI genome database at the time we conducted this study (https://www.ncbi.nlm.nih.gov (accessed on 3 August 2022)). 

Overall, a pan-genome of 2761 genes was identified in the isolates, of which 1509 genes were assigned a name and function whilst 1252 genes were annotated as hypothetical proteins (with unknown function). The core genome comprised 1941 genes and thereby formed 70% of the pan-genome, which is within the range of core genome sizes observed for other NAS from bovine mastitis [35]. The core genome decreased to 650 genes when performing a similar pan-genome analysis replacing the *S. rostri* DSM21968 strain with *S. microti* DSM 22147 as a reference, and it additionally dropped to 370 genes when using *S. muscae* ATCC 49910 as the reference. These observations suggest a somewhat high level of interspecies diversity among these three NAS species, although they are each other’s nearest relatives [10,11,13], and despite a reasonable level of gene conservation within *S. rostri*.

### 3.4. Virulence Factors, Antibiotic Resistance and Mobile Genetic Elements

To explore the pathogenicity of *S. rostri*, we evaluated the distribution of genes associated with virulence and antibiotic resistance. In this regard, we further looked for MGEs and discussed their potential association to virulence and antibiotic resistance. 

All 81 isolates and the *S. rostri* DSM 21968 strain carried two putative virulence genes: the ATP-dependent protease subunits *clpP* and *clpC* (Table S4). Overall, the Clp family of ATPases plays a crucial role in the folding, assembly, and degradation of proteins, and thereby maintenance of homeostasis [36]. While the *clpP* gene is well-conserved in most bacterial species, the *clpC* gene is usually found in Gram-positive bacteria specifically. The two genes can associate to form a proteolytic complex (i.e., ClpCP), which can regulate virulence in Gram-positive bacteria regarding, e.g., stress tolerance, biofilm formation, and motility [36,37]. However, in the present study, the *clpP* and *clpC* genes were not detected in the same contig for any of the isolates (nor in *S. rostri* DSM 21968 strain), and thereby they were presumably not forming such a complex; thus, their exact virulence properties in *S. rostri* remain uncertain. 

**Figure 1:**
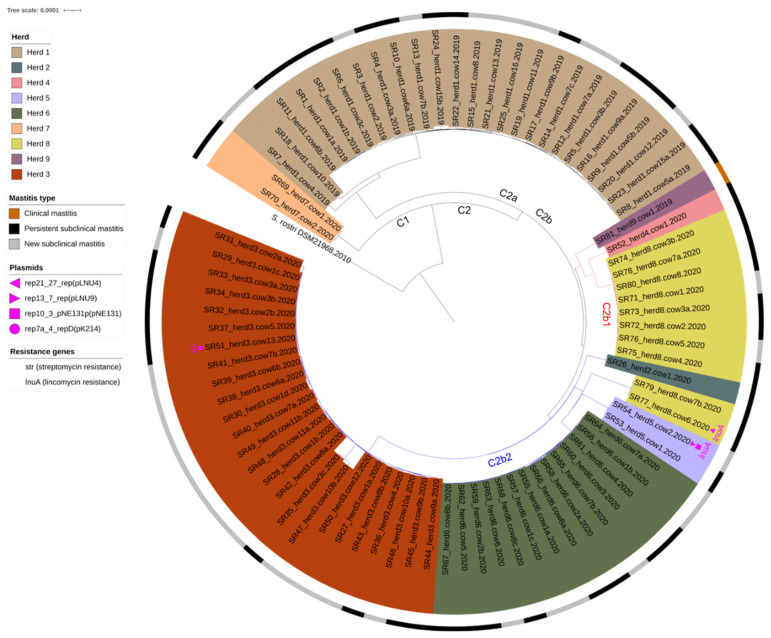
Maximum-likelihood phylogenetic tree based on the core genome of 81 *S. rostri* isolates from bovine mastitis and *S. rostri* DSM 21968 (GenBank Accession no. GCF002902145) as a reference (tree re-rooted from the reference). The test isolates were labeled from SR1-SR81 (S for “*Staphylococcus*”, R for “*rostri*”) and assigned a herd and cow number. In cows with *S. rostri* in >1 quarters, the isolates were additionally named a–d depending on the number of *S. rostri*-positive quarters as follows: “a” for the isolate from the *S. rostri*-positive quarter number 1; “b” for the positive quarter number 2; and so forth.

Three isolates were found to carry a resistance gene (Figure 1, Table S4). These genes were, respectively, *str* (encoding streptomycin resistance) carried by SR51_herd3.cow13, and *lnuA* (encoding lincomycin resistance) carried by SR54_herd5.cow2 and SR77_herd8.cow6. The resistant isolates were further found to carry at least one plasmid replicon in the same contig as the resistance gene (Figure 1, Table S4), which might indicate mediation of plasmid-borne resistance through horizontal gene transfer. The streptomycin resistant isolate, SR51_herd3.Cow13, yielded a rep7a_4_repD(pK214) replicon, which has previously been associated with streptomycin resistance [38]. The lincomycin resistant isolates, SR54_herd5_cow2 and SR77_herd8.cow6, carried replicon genes of rep13_7_rep(pLNU9) and rep21_27_rep(pLNU4), respectively. The lincosamide resistance plasmids were first recovered in bovine *S. chromogenes* SCM isolates in 2007 [39], but both SR54_herd5.cow2 and SR77_herd8.cow6 were found in pure cultures (Table S2). The SR54_herd5.cow2 isolate further carried a rep10_3_pNE131p1(pNE131) replicon, and finally the *S. rostri* DSM 21968 reference strain also yielded the rep13_7_rep(pLNU9) replicon although no resistance genes were observed here.

To summarize, a limited extent of known virulence and antibiotic resistance genes were found in the *S. rostri* isolates. We therefore speculated if other genes would be present with pathogenetic influence in *S. rostri.* To explore this, we evaluated the gene differences among the bovine isolates according to infection type, i.e., new SCM vs. persistent SCM and CM vs. SCM; additionally, differences in gene content between the bovine isolates and the porcine *S. rostri* DSM 21968 reference strain were evaluated. The latter, to explore the presence of genes potentially involved with host specificity. In this context, we did not predict any genes exclusively associated with SCM overall, or new SCM or persistent SCM (Table S3). 

**Figure 2:**
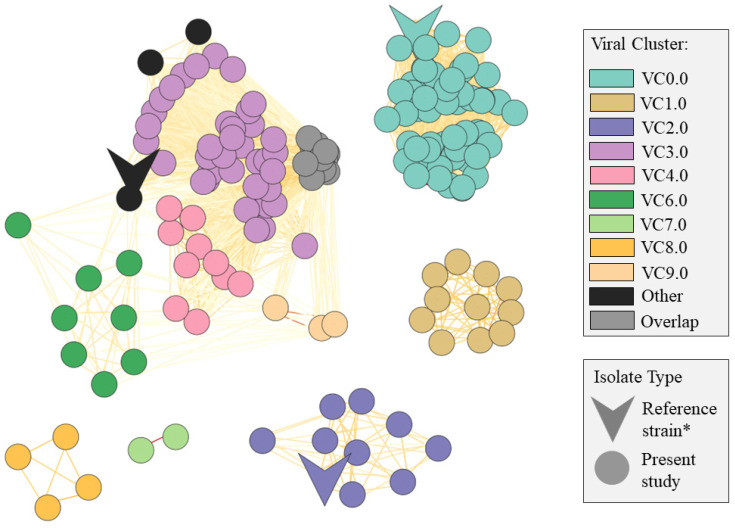
Network of viral clusters (VCs) representing the predicted phages among the *S. rostri* isolates. “Overlap”: phages sharing overlap with other phages from multiple VCs; “Other”: phages classified as singletons or outliers; “singletons”: phages with only a few or no gene similarity against other phages; “outliers”: phages with some genes shared with other phages, but not enough to be placed within a VC [28]. Reference strain*: the *S. rostri* DSM 21968.

Interestingly, 61 genes were found to be unique for the CM isolate (SR81_herd9.cow1) (Table S3). While 21/61 genes had an annotated function, mostly related to phage elements, the remaining 40/61 genes were annotated as hypothetical proteins which may be candidates for further examination on virulence properties. Furthermore, 26 genes were shared among all the 81 bovine isolates but were absent in the porcine *S. rostri* DSM 21968 strain (Table S3). Of these 26 genes, only 10 were loosely annotated as being involved in gene transfer. Taking this together, we speculated whether the *S. rostri* isolates were carrying phages with influence on (a) the pathogenicity of *S. rostri* and (b) the host-specificity of *S. rostri*. To explore this, we investigated the overall distribution of phages among the *S. rostri* population and evaluated their relation to each other via a viral clustering network (Figure 2). 

One to five phages were predicted for each of all the *S. rostri* isolates, with the exception of a single isolate from herd 3 (SR_36_herd3.cow6) where no phages were found. Overall, the phage prediction suggested nine different phage-species each distributed as a viral cluster (VC) (Figure 2). No phage was found in all the bovine study isolates as speculated, since the largest VC (VC0.0) covered 56 phages identified in isolates representing only 6/9 herds (herds 1, 2, 3, 4, 7, and 8) and furthermore in the *S. rostri* DSM 21968 strain. However, three VCs were found covering phages solely from bovine isolates (VC1.0, VC7.0, and VC8.0), thereby suggesting some bovine-unique phages for *S. rostri*. Within these three VCs, more herds (herd 1, 3, 8, and 9) were represented, although the VCs were all small (VC1.0 = 11 isolates, VC7.0 = 2 isolates, and VC8.0 = 4 isolates). Five phages were observed in the CM isolate (SR81_herd9.cow1), where four out of five clustered together with other phages from SCM isolates and/or the reference strain. The remaining 1/5 predicted phage was a singleton, i.e., was not part of a VC as it did not share gene similarity with any other of the predicted phages. This could demonstrate a phage with impact on the pathogenicity of *S. rostri,* but this needs further investigation.

### 3.5. Core genome Phylogeny

To further investigate the genetic relatedness among the *S. rostri* isolates, a phylogenetic tree was constructed based on the core genome alignment of the entire *S. rostri* population, including the porcine *S. rostri* DSM 21968 strain (Figure 1). Two overall lineages were found: one, consisting only of the *S. rostri* DSM 21968 strain and a second comprising all the 81 bovine isolates. The bovine isolates were distributed in two clades (C1 and C2) (with a clade referring to a grouping of isolates composed of a common ancestor and all the descendants from that common ancestor). The C1 clade consisted solely of the two isolates from herd 7 and the C2 clade of the remaining seventy-nine bovine isolates and eight herds. The C2 clade further displayed two subclades (C2a and C2b), with C2a covering all isolates from herd 1 (*n* = 25 isolates) and C2b covering the isolates from the remaining herds (herds 2, 3, 4, 5, 6, 8, 9) (*n* = 54 isolates). Of note, the isolates in C2 clustered together into smaller groups (subclades) according to their herd of origin, which indicates the presence of herd-specific *S. rostri* strains. 

Clade C2a, covering all isolates from herd 1 (*n* = 25), included a large subclade containing 22/25 isolates, which could demonstrate more *S. rostri* genotypes within herd 1. For this large subclade, the intra SNP value was 33 while it increased to 77 when including all isolates from herd 1 (the entire clade C2a). Clade C2b consisted of two subclades named clade C2b1 (red branches, *n* = 10 isolates) and C2b2 (blue branches, *n* = 44 isolates) (Figure 1). C2b1 included the CM isolate (SR81_herd9.cow1), the single isolate from herd 4 (SR52_herd4.cow1), and 8/10 isolates from herd 8, while C2b2 covered the single isolate from herd 2 (SR26_herd2.cow1) and all isolates from herds 3, 5, 6, and 2/8 isolates from herd 8 (Figure 1). The intra SNP for the entire C2b1 was 162, and it was 10 for the subclade consisting of 8/10 isolates from herd 8. The intra SNP for the entire C2b2 was 299, and it was 55 for the subclade containing all isolates from herd 3 (*n* = 25), 11 for the subclade including the two isolates from herd 5, and 6 for the subclade covering all isolates from herd 6 (*n* = 14). The clearly observed clustering pattern of the isolates according to herd of origin, combined with the low intra-herd SNPs, revealed that the *S. rostri* strains consisted of herd-specific clones. 

## 4. Discussion

This study presents the first characterization of *S. rostri* isolated from bovine mastitis. The isolates were initially identified as *S. microti* by MALDI-TOF MS, but reclassified as *S. rostri* following WGS and ANI analysis including the *S. microti* DSM 22147, *S. rostri* DSM 21968, and *S. muscae* ATCC 49910 as controls. These reference species were chosen due to previous studies demonstrating them to be the nearest relatives to each other [10,11,13]. Of note, we later found that *S. rostri* was not present in the applied MALDI-TOF MS spectrum library, which probably led to the misidentification of the isolates at first. We therefore accentuate the importance of integrating spectra of novel bovine strains to MALDI-TOF MS libraries to enhance species identification and future diagnostic performance based on MALDI-TOF MS. 

To explore the pathogenesis of *S. rostri* as a possible mastitis agent, we investigated the distribution of virulence and antibiotic resistance genes. Only two putative virulence genes were detected among the *S. rostri* isolates (*clpP* and *clpC*). Despite the fact that several virulence genes have been observed among NAS overall, some studies report virulence genes being less frequent among NAS compared to the major pathogen *S. aureus* [4,35], and that the presence, or absence, of virulence genes among NAS does not directly correlate with the severity of mastitis they might cause [35]. We performed similar observations as the latter suggestion, since the identified virulence genes were part of the core genome, hence no differences in the presence, or absence, of virulence genes were found according to the type of mastitis (CM vs. SCM or new SCM vs. persistent SCM) in our study.

In Denmark, bovine mastitis is the main indication for antibiotic usage in adult dairy cows (cows > 1 year old), and beta-lactamase sensitive penicillins is the class counting for most of the antibiotic usage in this regard [40]. However, no resistance genes encoding penicillin resistance were found in any of the isolates in this study. Overall, the occurrence of genetic antibiotic resistance among the isolates was low as only three isolates were carrying a resistance gene encoding streptomycin or lincomycin resistance, respectively. Antibiotic resistance trends among *S. rostri* have been studied previously, but for isolates with origins other than bovine mastitis and the observed resistance levels seem to vary. Stegmann and associates found *S. rostri* isolates from pigs resistant towards tetracycline, penicillin, streptomycin, clindamycin, erythromycin, and trimethoprim [41]. Vanderhaeghen and associates reported two methicillin-resistant *S. rostri* from pigs. These isolates were positive for the *mecA* gene, but they were beta-lactam-susceptible phenotypically [42]. Locatelli and associates found *S. rostri* from dairy water buffaloes susceptible to all antibiotics tested [43]. Finally, Wuytack and associates studied the resistance trends among bovine fecal *S. rostri* isolates and found all isolates expressing both phenotypic and genotypic resistance towards beta-lactams [16]. We did not perform phenotypic resistance determination in the present study due to funding limitations and therefore we cannot know if the genotypic resistance profiles detected are expressed phenotypically. But since antibiotic resistance has indeed been reported among *S. rostri* previously [16,41], surveilling phenotypic resistance trends among *S. rostri* from bovine mastitis may become a relevant topic in future research. 

To summarize the virulence and resistance gene distribution, a limited distribution of virulence and antibiotic resistance genes were found among the *S. rostri* isolates. However, it is worth mentioning that a possible explanation for these observations could be due to the sequencing strategy applied, i.e., 2. generation (short-read) sequencing which, unlike 3. generation (long-read) sequencing, provides contigs instead of a closed genome; hence, some genes might not be detected due to fragment overlap or sequence gaps.

Regarding the classification of mastitis, NAS are often involved in persistent SCM, but the genetic factors behind this capability are still not clear. Importantly, in this study, persistent SCM isolates were classified as such, when isolates derived from cows with an increased SCC measured for at least 2/3 of the last RYK recordings (corresponding to approximately the last 2–3 months). By this, the historic SCC data served as an indicator of SCM persistency, but whether the previously measured increased SCCs were due to one continuous, possibly intermittent infection, or to several new infections remains unclear. Hence, it is unknown if the persistent SCM was due to *S. rostri* alone, another pathogen than *S. rostri*, or by the combined presence of *S. rostri* and other pathogen(s) over time. Therefore, a possible reason for not finding any genetic differences explicitly among the group of new versus persistent SCM isolates in this study might be that the persistent SCM cases were not related to *S. rostri* alone. Likewise, our study cannot exclude that cow-factors are more predictive for the outcome of acute versus persistent SCM, nor for the outcome of SCM versus CM when *S. rostri* is present in the udder, as cow-factors have not been included in our study. Also, the low number of clinical isolates hampers the comparison between CM versus SCM.

Considering the possible transmission routes, the core genome phylogeny combined with the low intra SNPs measured suggested the presence of herd-specific clones. Such findings are of epidemiological importance and imply that each herd probably had its own introductory source(s). In addition, such findings may suggest two possible scenarios: (a) that *S. rostri* was infecting the cows separately from a single unknown source specific for each herd, or (b) that *S. rostri* can adapt to the bovine mammary gland following cow-to-cow transmission, and thereby that *S. rostri* can act as a contagious mastitis pathogen. 

Reported findings of *S. rostri* from domestic animals and in general are very sparse, although *S. rostri* has been described as a natural colonizer of the nasal cavities in healthy pigs [41]. Hence, one could speculate if the bovine *S. rostri* were acquired from a porcine-related source. However, none of the herds in the present study were performing pig husbandry at the time this study was conducted, even though herds 1, 3, 5, and 6 were previously farming pigs according to the national Central Husbandry Register (CHR) database [44]. But for these four herds, the pig husbandry ended at least a decade ago. Also, the phylogenetic analysis showed that the two isolates from herd 7 (SR69_herd7.cow1 and SR70_herd7.cow2) were closest related to the porcine *S. rostri* DSM 21968 reference (Figure 1), but according to the CHR database, herd 7 was never associated with pig farming. Taking this together, pigs cannot be pointed out as the direct source of introduction due to spill-over to the herd environment from previous pig husbandry. However, it cannot be precluded that the introduction sources were related to some spill-over related to pigs in another manner (e.g., transmission from other hosts associated with pigs such as farm staff.). In addition, the isolates from herd 8 clustered separately in two different clades (C2b1 and C2b2) suggesting more genotypes within herd 8 as similar for herd 1 (Figure 1). This could reflect the introduction and spread of *S. rostri* more than once, e.g., due to animal trading, new farm personnel, etc. According to the CHR database, animal movement did take place during the last decade for all herds included in this study, but not between any of the test herds. Nor did any of the test herds move animals to or from the same herds as the other test herds.

Discussing the pathogenetic potential of *S. rostri*, Wuytack and associates previously found *S. rostri* as the most prevalent NAS among fecal samples from healthy dairy cows, hypothesizing *S. rostri* to act as an indicator of fecal contamination to bulk tank milk like *E. coli* [16]. We cannot preclude *S. rostri* as an environmental contaminator in our milk samples. However, we found 24/80 (30%) of the *S. rostri* isolates from SCM as pure cultures, and of note, the single *S. rostri* isolate from CM as a pure culture as well, which indicate the ability of *S. rostri* to act as a mastitis pathogen itself. Furthermore, we identified 12 additional *S. rostri* isolates, which were excluded from the overall study analysis, as they were found in samples with ≥2 other bacterial species, hence classified as contaminated. These isolates were from some of the present study herds (herds 1, 3, 4, and 8) and a single isolate was from a herd not represented in the present study (herd 10). When performing a pan-genome analysis followed by a phylogenetic tree of the core genome alignment after adding these 12 additional *S. rostri* isolates, we did not observe any distinctive changes in the clustering pattern (i.e., the contamination-related isolates clustered together with the non-contamination-related isolates according to herd of origin). Furthermore, no genes were found to be unique for the group of contamination-related *S. rostri* isolates (Table S3). Accordingly, the contaminated samples support the conclusion that *S. rostri* is farm-specific. Therefore, it is not possible to discern whether *S. rostri* in mixed culture samples are present due to contamination of the milk sample or not. Yet, it is expected that if a milk sample is contaminated, there would be >2 bacterial species present [18]. Hence, the high number of mixed culture samples (i.e., 2 bacterial species) (*n* = 57) versus the lower number of contaminated samples (*n* = 12), that contain *S. rostri* indicates that *S. rostri* is more likely to be related to the milk rather than to contamination of it. If *S. rostri* should be considered a contaminant rather than a pathogen, we would expect more contaminated samples and fever mixed and pure cultures. 

Last but not least, when *S. rostri* was found with another pathogen (mixed samples) there was no pattern of what pathogens were found together with *S. rostri*, except that the other pathogen was most often another NAS. Hence, there was no indication that *S. rostri* was merely accompanying flora of more important pathogens.

Summing up on the pathogenetic potential, the relatively high proportions of pure- and mixed culture samples, the relatively low proportion of contaminated samples, and the lack of pattern in the mixed samples indicates that *S. rostri* should be considered at least an opportunistic mastitis pathogen.

## 5. Conclusions

The present study reports the finding and characterization of 81 *S. rostri* isolates from bovine mastitis. The isolates were identified as *S. microti* by MALDI-TOF MS followed by WGS and later classified as *S. rostri* by ANI analysis. The isolates came from 52 cows from nine Danish dairy herds. Overall, the results showed limited presence of virulence and resistance genes. All isolates carried two putative genes involved with virulence, *clpP* and *clpC*, and three isolates carried a single resistance gene encoding streptomycin or lincomycin resistance, respectively. A pan-genome analysis revealed an overall high gene conservation in *S. rostri,* although a core genome phylogeny analysis showed that the *S. rostri* isolates consisted of herd-specific clones. Also, we observed that *S. rostri* was found only rarely in contaminated samples. Altogether, our study raises the hypothesis that *S. rostri* might act as a mastitis pathogen and that it persists on farms either via cow-to-cow transmission or via unknown environmental niche(s).

## Figures and Tables

**Table 1:** Overview of *S. rostri* isolates from bovine mastitis and the number of cows infected by it from 2019 to 2020.

Herd	*n* Isolates	*n* Animals	*n* Animals One Quarter	*n* Animals Two Quarters	*n* Animals Three Quarters	*n* Animals All Quarters
herd 1	25	16	9	5	2	-
herd 2	1	1	1	-	-	-
herd 3	25	13	4	7	1	1
herd 4	1	1	1	-	-	-
herd 5	2	2	2	-	-	-
herd 6	14	8	4	2	2	-
herd 7	2	2	2	-	-	-
herd 8	10	8	6	2	-	-
herd 9	1	1	1	-	-	-

**Table 2:** Bacterial species found in mixed cultures with *S. rostri* isolates. From a total of 57 milk samples, 16 different species were identified in combination with *S. rostri*.

Bacterial Species	*n*
*Staphylococcus simulans*	14
*Staphylococcus epidermidis*	9
*Staphylococcus haemolyticus*	7
*Corynebacterium amycolatum*	6
*Lactococcus garvieae*	4
*Aerococcus viridans*	3
*Staphylococcus chromogenes*	3
*Streptococcus gallolyticus*	2
*Streptococcus uberis*	1
*Streptococcus canis*	1
*Staphylococcus muscae*	1
*Enterococcus faecalis*	1
*Escherichia coli*	1
*Lactococcus lactis*	1
*Citobacter koseri*	1
*Kocuria rhizophila*	1
“No ID”	1

## Data Availability

Genomes from this study have been deposited with NCBI under BioProject no. PRJNA988321. Datasets generated and analyzed during the study, which are not provided as Supplementary Materials, are available from the corresponding author upon reasonable request.

## Supplementary Materials

The following supporting information can be downloaded at: https://www.mdpi.com/article/10.3390/vetsci10090530/s1, Table S2. Overview of *S. rostri* isolates from dairy cows with mastitis; Table S3. Query_pan_genome results; Table S4. Presence of virulence, resistance and plasmid genes; Figure S1. Average Nucleotide Identity of *S. rostri*; Table S1. Assembly metrics of *S. rostri* isolates; Figure S2. Core genome phylogeny of 93 *S. rostri* isolates.
