# Supplementary material for: Comparative Genomics of Staphylococcus rostri, an Undescribed Bacterium Isolated from Dairy Mastitis
Source: Vet Sci. 2023 Aug 22;10(9):530. doi: 10.3390/vetsci10090530 (PMC10534715; doi:10.3390/vetsci10090530)

Figure S1: Average Nucleotide Identity (%) of *S. rostri* isolates **a)** with three NAS reference strains: *S. microti* DSM22147 (NCBI assembly accession GCA002902635), *S. rostri* DSM21968 (NCBI assembly accession GCF002902145), and *S. muscae* ATCC49910 (NCBI assembly accession GCF_003019275), and **b)** solely with the *S. rostri* DSM21968 (NCBI assembly accession GCF002902145).

a)

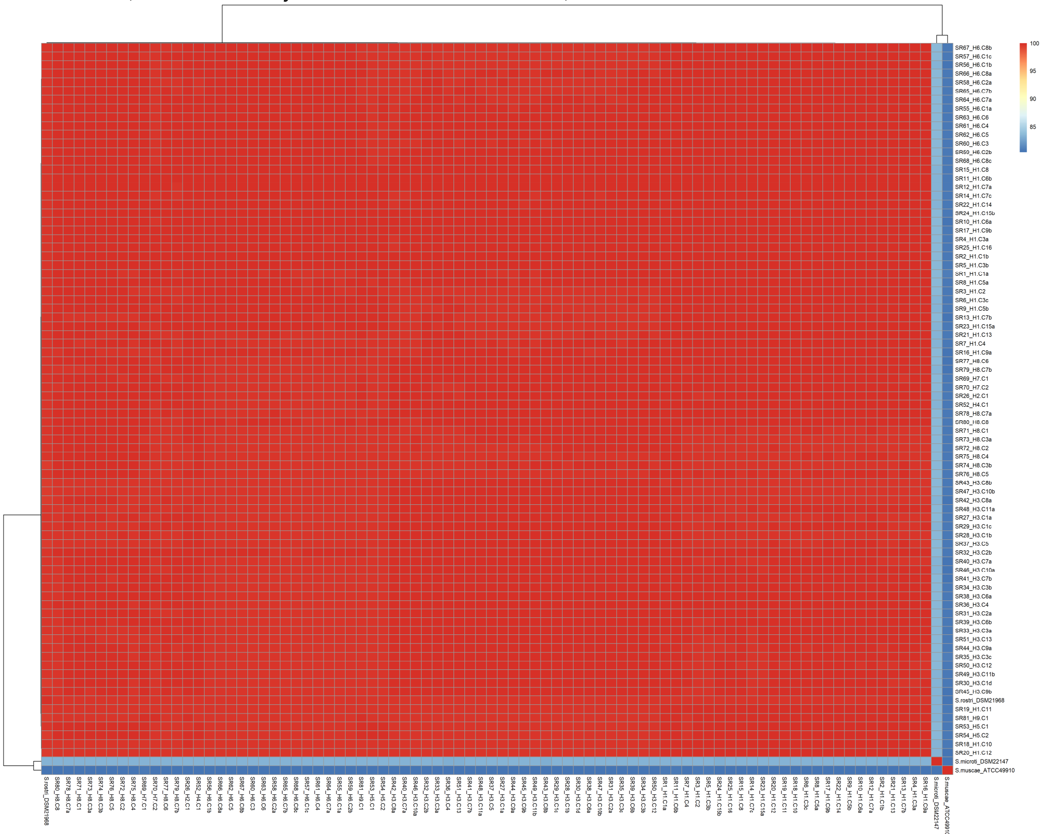

b)

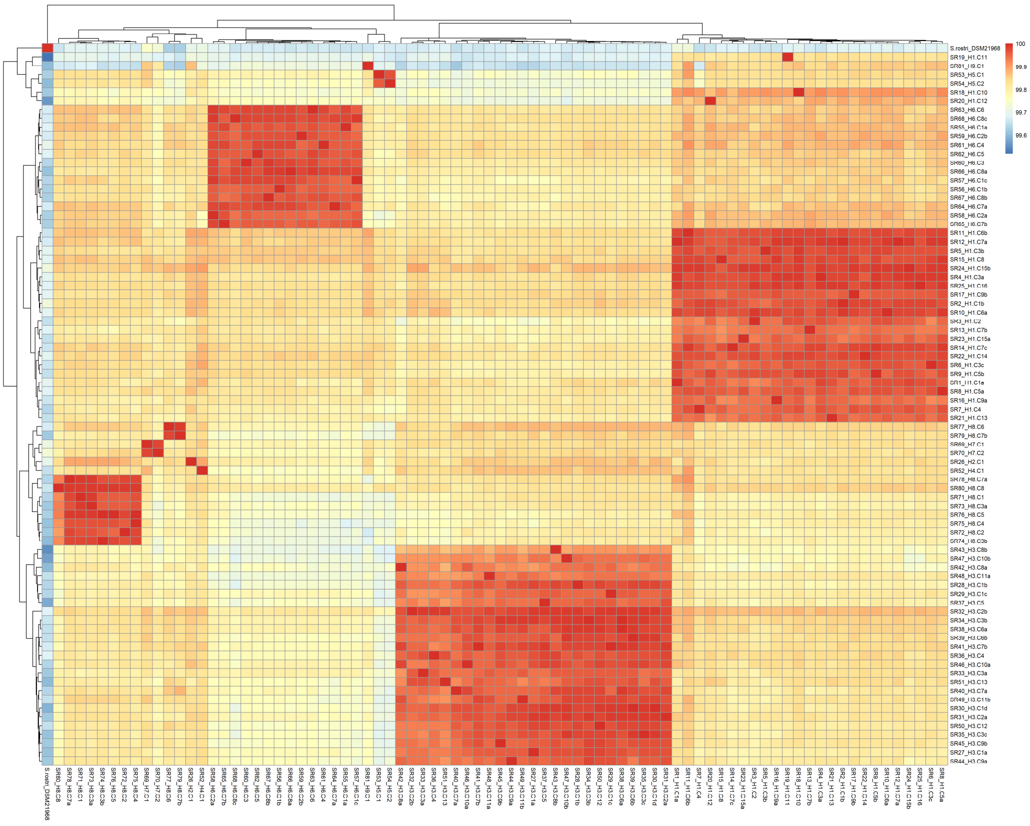

Supplement: Supplementary file 1 [file vetsci-10-00530-s001.zip › Figure S1 Average Nucleotide Identity of S. rostri isolates.pdf]
