# Supplementary material for: Comparative Genomics of Staphylococcus rostri, an Undescribed Bacterium Isolated from Dairy Mastitis
Source: Vet Sci. 2023 Aug 22;10(9):530. doi: 10.3390/vetsci10090530 (PMC10534715; doi:10.3390/vetsci10090530)
Supplement: Supplementary file 1 [file vetsci-10-00530-s001.zip › Table S2 Overview of S. rostri isolates from dairy cows with mastitis.pdf]

Table S2. Overview of *S. rostri* isolates from dairy cows with mastitis.

Isolate designation	Collection Date	Gland*	Type of mastitis	Culture Type	n different species found in mixed culture	Finding A, B or C**
SR1_H1C1a	October 2019	RR	Persistent SCM	Mixed	2	*S. rostri* (A)<br>*Streptococcus uberis* (B)
SR2_H1C1b	October 2019	LR	Persistent SCM	Mixed	2	*S. rostri* (A)<br>*Staphylococcus muscae* (B)
SR3_H1C2	October 2019	RF	New SCM	Mixed	2	*S. rostri* (A)<br>*Staphylococcus epidermidis* (B)
SR4_H1C3a	October 2019	RF	Persistent SCM	Mixed	2	*S. rostri* (A)<br>*Staphylococcus epidermidis* (B)
SR5_H1C3b	October 2019	LF	Persistent SCM	Mixed	2	*Staphylococcus simulans* (A)<br>*S. rostri* (B)
SR6_H1C3c	October 2019	RR	Persistent SCM	Mixed	2	*S. rostri* (A)<br>*Corynebacterium amycolatum* (B)
SR7_H1C4	October 2019	LR	New SCM	Mixed	2	*Staphylococcus haemolyticus* (A)<br>*S. rostri* (B)
SR8_H1C5a	October 2019	RF	Persistent SCM	Pure	1	
SR9_H1C5b	October 2019	LF	Persistent SCM	Mixed	2	*Staphylococcus simulans* (A)<br>*S. rostri* (B)
SR10_H1C6a	October 2019	RR	Persistent SCM	Mixed	2	*S. rostri* (A)<br>*Staphylococcus haemolyticus* (B)
SR11_H1C6b	October 2019	LR	Persistent SCM	Mixed	2	*S. rostri* (A)<br>*Lactococcus garvieae* (B)
SR12_H1C7a	October 2019	RF	New SCM	Mixed	2	*Staphylococcus haemolyticus* (A)<br>*S. rostri* (B)
SR13_H1C7b	October 2019	RR	New SCM	Mixed	2	*Staphylococcus simulans* (A)<br>*S. rostri* (B)

SR14_H1C7c	October 2019	LR	New SCM	Mixed	2	*Staphylococcus simulans* (A)<br>*S. rostri* (B)
SR15_H1C8	October 2019	RR	New SCM	Pure	1	
SR16_H1C9a	October 2019	LF	Persistent SCM	Mixed	2	*Staphylococcus simulans* (A)<br>*S. rostri* (B)
SR17_H1C9b	October 2019	LR	Persistent SCM	Mixed	2	*Staphylococcus haemolyticus* (A)<br>*S. rostri* (B)
SR18_H1C10	October 2019	LF	New SCM	Pure	1	
SR19_H1C11	October 2019	RR	New SCM	Pure	1	
SR20_H1C12	October 2019	LR	New SCM	Mixed	2	*Staphylococcus simulans* (A)<br>*S. rostri* (B)
SR21_H1C13	October 2019	LF	New SCM	Mixed	2	*S. rostri* (A)<br>*Aerococcus viridans* (B)
SR22_H1C14	October 2019	RF	Persistent SCM	Mixed	2	*Staphylococcus simulans* (A)<br>*S. rostri* (B)
SR23_H1C15a	October 2019	RR	Persistent SCM	Mixed	2	*Lactococcus garvieae* (A)<br>*S. rostri* (B)
SR24_H1C15b	October 2019	LR	Persistent SCM	Pure	1	
SR25_H1C16	October 2019	RF	New SCM	Mixed	2	*S. rostri* (A)<br>*Corynebacterium amycolatum* (B)
SR26_H2C1	January 2020	LR	Persistent SCM	Mixed	2	*S. rostri* (A)<br>*Corynebacterium amycolatum* (B)
SR27_H3C1a	January 2020	RF	New SCM	Mixed	2	*S. rostri* (A)<br>*Enterococcus faecalis* (B)
SR28_H3C1b	January 2020	LF	New SCM	Mixed	2	*S. rostri* (A)<br>*Escherichia coli* (B)
SR29_H3C1c	January 2020	RR	New SCM	Mixed	2	*Staphylococcus epidermidis* (A)<br>*S. rostri* (B)
SR30_H3C1d	January	LR	New	Mixed	2	*S. rostri* (A)

	2020		SCM			*Lactococcus garvieae* (B)
SR31_H3C2a	January 2020	LF	Persistent SCM	Mixed	2	*S. rostri* (A)<br>*Staphylococcus chromogenes* (B)
SR32_H3C2b	January 2020	RR	Persistent SCM	Mixed	2	*S. rostri* (A)<br>*Staphylococcus chromogenes* (B)
SR33_H3C3a	January 2020	RF	Persistent SCM	Mixed	2	*S. rostri* (A)<br>*Staphylococcus epidermidis* (B)
SR34_H3C3b	January 2020	LF	Persistent SCM	Mixed	2	*Staphylococcus simulans* (A)<br>*S. rostri* (B)
SR35_H3C3c	January 2020	RR	Persistent SCM	Mixed	2	*Staphylococcus epidermidis* (A)<br>*S. rostri* (B)
SR36_H3C4	January 2020	RR	Persistent SCM	Mixed	2	*Staphylococcus epidermidis* (A)<br>*S. rostri* (B)
SR37_H3C5	January 2020	LF	Persistent SCM	Mixed	2	*Staphylococcus chromogenes* (A)<br>*S. rostri* (B)
SR38_H3C6a	January 2020	LF	New SCM	Mixed	2	*S. rostri* (NA)<br>*Staphylococcus simulans* (NA)
SR39_H3C6b	January 2020	LR	New SCM	Mixed	2	*S. rostri* (NA)<br>*Staphylococcus simulans* (NA)
SR40_H3C7a	January 2020	RF	New SCM	Pure	1	
SR41_H3C7b	January 2020	RR	New SCM	Mixed	2	*S. rostri* (NA)<br>*Aerococcus viridans* (NA)
SR42_H3C8a	January 2020	LF	New SCM	Pure	1	
SR43_H3C8b	January 2020	RR	New SCM	Mixed	2	*S. rostri* (NA)<br>*Staphylococcus epidermidis* (NA)
SR44_H3C9a	January 2020	RF	Persistent SCM	Mixed	2	*S. rostri* (NA)<br>*Lactococcus garvieae* (NA)
SR45_H3C9b	January 2020	LF	Persistent SCM	Mixed	2	*S. rostri* (A)<br>*Staphylococcus simulans* (B)
SR46_H3C10a	January 2020	RF	New SCM	Pure	1	

SR47_H3C10b	January 2020	RR	New SCM	Pure	1	
SR48_H3C11a	January 2020	RF	New SCM	Mixed	2	*S. rostri* (NA)<br>*Staphylococcus haemolyticus* (NA)
SR49_H3C11b	January 2020	LF	New SCM	Pure	1	
SR50_H3C12	January 2020	RF	New SCM	Mixed	2	*S. rostri* (NA)<br>*Staphylococcus haemolyticus* (NA)
SR51_H3C13	January 2020	LR	Persistent SCM	Pure	1	
SR52_H4C1	January 2020	LF	Persistent SCM	Mixed	2	*S. rostri* (NA)<br>*Staphylococcus epidermidis* (NA)
SR53_H5C1	January 2020	RR	Persistent SCM	Pure	1	
SR54_H5C2	January 2020	RR	New SCM	Pure	1	
SR55_H6C1a	February 2020	RF	New SCM	Mixed	2	*No ID* (A), *S. rostri* (B)
SR56_H6C1b	February 2020	RR	New SCM	Pure	1	
SR57_H6C1c	February 2020	LR	New SCM	Pure	1	
SR58_H6C2a	February 2020	RF	Persistent SCM	Mixed	2	*S. rostri* (A)<br>*Streptococcus gallolyticus* (B)
SR59_H6C2b	February 2020	LF	Persistent SCM	Pure	1	
SR60_H6C3	February 2020	LF	Persistent SCM	Mixed	2	*Staphylococcus simulans* (A)<br>*S. rostri* (B)
SR61_H6C4	February 2020	LR	Persistent SCM	Pure	1	
SR62_H6C5	February 2020	LF	Persistent SCM	Mixed	2	*S. rostri* (A)<br>*Staphylococcus simulans* (B)
SR63_H6C6	February	RR	New	Pure	1	

	2020		SCM			
SR64_H6C7a	February 2020	RF	New SCM	Mixed	2	*S. rostri* (A)<br>*Streptococcus gallolyticus* (B)
SR65_H6C7b	February 2020	RR	New SCM	Mixed	2	*S. rostri* (A)<br>*Staphylococcus simulans* (B)
SR66_H6C8a	February 2020	LR	Persistent SCM	Mixed	2	*Lactococcus lactis* (A)<br>*S. rostri* (B)
SR67_H6C8b	February 2020	RF	Persistent SCM	Pure	1	
SR68_H6C8c	February 2020	RR	Persistent SCM	Mixed	2	*Citobacter koseri* (A)<br>*S. rostri* (B)
SR69_H7C1	April 2020	RF	Persistent SCM	Mixed	2	*S. rostri* (A)<br>*Kocuria rhizophila* (B)
SR70_H7C2	April 2020	LR	Persistent SCM	Pure	1	
SR71_H8C1	May 2020	RF	New SCM	Mixed	2	*S. rostri* (A)<br>*Corynebacterium amycolatum* (B)
SR72_H8C2	May 2020	LR	Persistent SCM	Pure	1	
SR73_H8C3a	May 2020	RF	Persistent SCM	Mixed	2	*Streptococcus canis* (A)<br>*S. rostri* (B)
SR74_H8C3b	May 2020	LF	Persistent SCM	Mixed	2	*S. rostri* (A)<br>*Aerococcus viridans* (B)
SR75_H8C4	May 2020	RF	Persistent SCM	Mixed	2	*S. rostri* (A)<br>*Staphylococcus haemolyticus* (B)
SR76_H8C5	May 2020	RF	New SCM	Mixed	2	*Staphylococcus epidermidis* (A)<br>*S. rostri* (B)
SR77_H8C6	May 2020	RR	Persistent SCM	Pure	1	
SR78_H8C7a	May 2020	LF	Persistent SCM	Mixed	2	*Corynebacterium amycolatum* (A)<br>*S. rostri* (B)
SR79_H8C7b	May 2020	RR	Persistent SCM	Mixed	2	*S. rostri* (A)<br>*Corynebacterium amycolatum* (B)

SR80_H8C8	May 2020	LR	Persistent SCM	Pure	1	
SR81_H9C1	November 2019	NA	CM	Pure	1	

Gland*: Right front (RF), left front (LF), right rear (RR), and left rear (LR). Finding A, B, or C**: visual quantity of colonies grown on blood agar in mixed cultures; A=more, C=less. Not available (NA) is stated when even distribution of different species was found in the mixed cultures.
