# Supplementary figures and images for: Comparative Genomics of Staphylococcus rostri, an Undescribed Bacterium Isolated from Dairy Mastitis
Source: Vet Sci. 2023 Aug 22;10(9):530. doi: 10.3390/vetsci10090530 (PMC10534715; doi:10.3390/vetsci10090530)

Tree scale: 0.0001

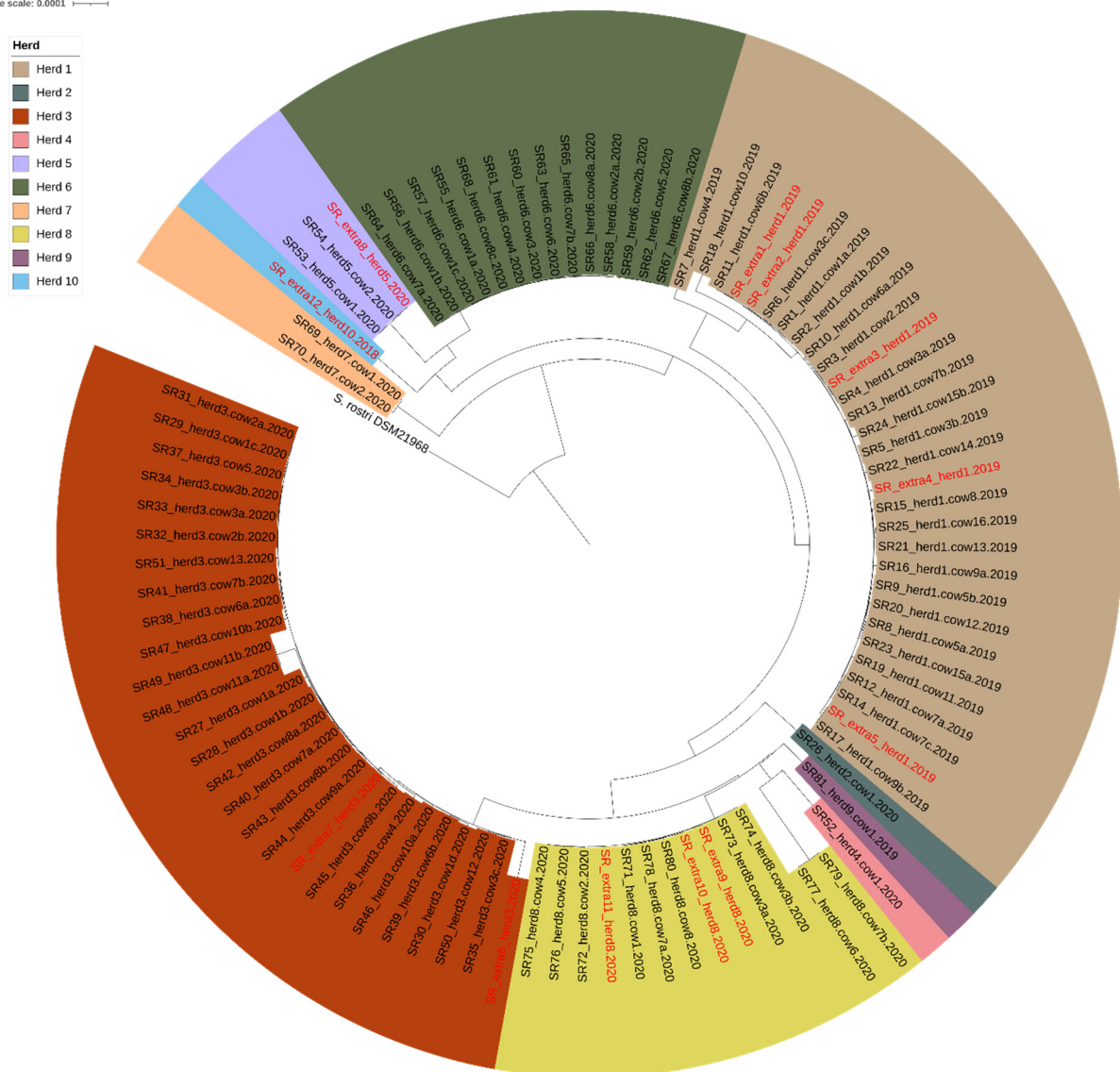

Supplement: Supplementary file 1 [file vetsci-10-00530-s001.zip › Figure S2 Core genome phylogeny of 93 S. rostri isolates.pdf]
